# Adipose tissue IL‐18 production is independent of caspase‐1 and caspase‐11

**DOI:** 10.1002/iid3.1241

**Published:** 2024-04-17

**Authors:** Luis Román‐Domínguez, Jonathan Salazar‐León, Karla F. Meza‐Sosa, Leonor Pérez‐Martínez, Gustavo Pedraza‐Alva

**Affiliations:** ^1^ Laboratorio de Neuroinmunobiología, Departamento de Medicina Molecular y Bioprocesos, Instituto de Biotecnología Universidad Nacional Autónoma de México Cuernavaca, Morelos Mexico; ^2^ Present address: Laboratorio de Neurobioquímica y Conducta Instituto Nacional de Neurología y Neurocirugía Manuel Velasco Suarez Ciudad de Méxicio Mexico

**Keywords:** adipose tissue, animals, cytokines, diabetes, inflammation, macrophage, molecules

## Abstract

**Background:**

Inflammation in adipose tissue, resulting from imbalanced caloric intake and energy expenditure, contributes to the metabolic dysregulation observed in obesity. The production of inflammatory cytokines, such as IL‐1β and IL‐18, plays a key role in this process. While IL‐1β promotes insulin resistance and diabetes, IL‐18 regulates energy expenditure and food intake. Previous studies have suggested that caspase‐1, activated by the Nlrp3 inflammasome in response to lipid excess, mediates IL‐1β production, whereas activated by the Nlrp1b inflammasome in response to energy excess, mediates IL‐18 production. However, this has not been formally tested.

**Methods:**

Wild‐type and caspase‐1‐deficient Balb/c mice, carrying the Nlrp1b1 allele, were fed with regular chow or a high‐fat diet for twelve weeks. Food intake and mass gain were recorded weekly. At the end of the twelve weeks, glucose tolerance and insulin resistance were evaluated. Mature IL‐18 protein levels and the inflammatory process in the adipose tissue were determined. Fasting lipid and cytokine levels were quantified in the sera of the different experimental groups.

**Results:**

We found that IL‐18 production in adipose tissue is independent of caspase‐1 activity, regardless of the metabolic state, while Nlrp3‐mediated IL‐1β production remains caspase‐1 dependent. Additionally, caspase‐1 null Balb/c mice did not develop metabolic abnormalities in response to energy excess from the high‐fat diet.

**Conclusion:**

Our findings suggest that IL‐18 production in the adipose tissue is independent of Nlrp3 inflammasome and caspase‐1 activation, regardless of caloric food intake. In contrast, Nlrp3‐mediated IL‐1β production is caspase‐1 dependent. These results provide new insights into the mechanisms underlying cytokine production in the adipose tissue during both homeostatic conditions and metabolic stress, highlighting the distinct roles of caspase‐1 and the Nlrp inflammasomes in regulating inflammatory responses.

## Graphical abstract

Our findings challenge previous notions about cytokine production in the adipose tissue, revealing independent pathways for IL‐1β and IL‐18. Nlrp1b inflammasome mediates caspase‐1‐independent IL‐18 production, while IL‐1β maturation by the Nlrp3 inflammasome remains caspase‐1 dependent.

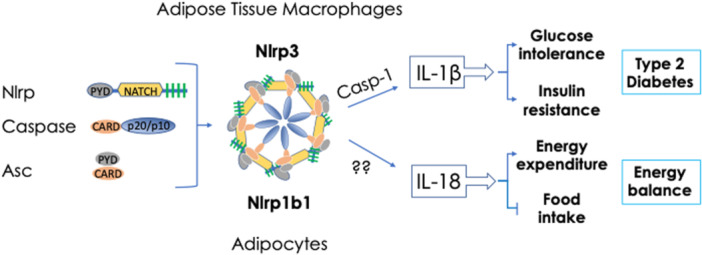

## 1 BACKGROUND

Obesity is characterized by the accumulation of excessive lipids in adipose tissue due to high caloric intake and low energy expenditure.^1^ This condition leads to metabolic abnormalities such as insulin resistance, glucose intolerance, and dyslipidemia.^2^ Chronic inflammation in adipose tissue plays a significant role in promoting these metabolic alterations. The inflammatory response is initiated by activated macrophages in response to metabolites released by stressed adipocytes and the uptake of lipids during phagocytosis of dead adipocytes, resulting in the production of inflammatory cytokines, including IL‐1β and IL‐18.^3^, ^4^ It has been believed that the Nlrp3 inflammasome, activated in response to lipid excess, recruits and activates caspase‐1 to produce both IL‐1β and IL‐18.^5^, ^6^ In contrast, the Nlrp1 inflammasome appears to have a protective role. Mice deficient in the Nlrp1 receptor exhibit spontaneous weight gain and metabolic abnormalities, which are exacerbated by a high‐fat diet (HFD).^7^ Interestingly, this phenotype correlates with reduced levels of IL‐18 in adipose tissue. Recent studies have shown that IL‐18 promotes energy expenditure and regulates energy intake.^8^, ^9^, ^10^ In mice, there are three Nlrp1 genes (Nlrp1a, Nlrp1b, and Nlrp1c), and the Nlrp1b gene is polymorphic, with five different alleles.^11^ Our recent work has demonstrated that the Nlrp1b1 allele‐encoded inflammasome promotes IL‐18 production in adipose tissue, protecting obese mice from metabolic abnormalities.^12^ Our findings support previous observations showing that Balb/c mice, which express the Nlrp1b1 allele and produce higher levels of IL‐18 in adipose tissue, are resistant to developing metabolic abnormalities when fed an HFD, unlike C57BL/6 mice carrying the Nlrp1b2 allele with low IL‐18 levels.^13^ Interestingly, in C57BL/6 transgenic mice expressing the Nlrp1b1 inflammasome, increased IL‐18 levels but reduced caspase‐1 activity in adipose tissue were observed when fed an HFD, while obese C57BL/6 wild‐type mice showed low IL‐18 levels but increased active caspase‐1 levels.^12^ Based on these findings, we hypothesized that Nlrp1b1‐mediated IL‐18 production in adipose tissue is independent of caspase‐1.

In this study, we demonstrate that caspase‐1‐deficient Balb/c mice, carrying the protective Nlrp1b1 allele, produce mature IL‐18 in the adipose tissue when fed an HFD, while IL‐1β levels in circulation are reduced. Additionally, caspase‐1 null Balb/c mice did not develop metabolic abnormalities in response to energy excess from the HFD. These results indicate that the Nlrp1b inflammasome produces IL‐18 independently of caspase‐1, while Nlrp3‐mediated IL‐1β production is caspase‐1 dependent. Our findings suggest that IL‐18 production in the adipose tissue is independent of Nlrp3 inflammasome and caspase‐1 activation, regardless of caloric food intake.

## 2 METHODS

### 2.1 Animal subjects

Two months old male wild‐type Balb/c and *Caspase‐1* ^−/−^/*Caspase11* ^−/−^ Balb/c mice were maintained in ventilated racks at the Instituto de Biotecnología animal facility. After genotyping animals were assigned to the different experimental groups. A total of 6 animals per experimental group were used, and three animals per cage were maintained during the experimentation. Mice were fed with regular chow (2018SX; Harlan Teklad Global, ND) or with an HFD (D12492, Research diets, HFD) and water ad libitum. Food intake and body mass were recorded weekly for 3 months. All experimental procedures were approved by the Bioethical committee of the Instituto de Biotecnología, Universidad Nacional Autónoma de México, following Mexican and International guidelines. Protocol 408.

### 2.2 Antibodies

The anti‐IL‐18 antibody (sc‐7954) was obtained from Santa Cruz Biotechnology. The anti‐p‐HSL^ser660^ (46804), the anti‐HSL (4107), and the anti‐GAPDH antibodies (14C10) were from Cell Signaling Technology.

### 2.3 Glucose and insulin tolerance tests

Animals were starved for 6hr. For the Glucose tolerance test (GTT) animals received intraperitoneal glucose (1.8 mg), while for the Insulin sensitivity test (ITT) animals received recombinant insulin (1 mU, Humulin® R) per gram of body mass. The blood glucose concentration was determined at 0, 15, 30, 60, and 120 min after injection using a One touch® meter. The area under the curve was calculated following Tai's formula:

Area=12∑ι=1nXι−1(ϒι−1+ϒι),

where *X*_1_  =  Glucose (mg/dL) and (Υι − 1 + Υι)(Υι − 1 + Υι) = Time (0, 15, 30, 60, 120 min).^14^

### 2.4 Tissue preparation

Retro peritoneal visceral adipose tissues were fixed in 4% paraformaldehyde in phosphate‐buffered saline (PBS) overnight at 4°C, then incubated in 30% sucrose in PBS overnight at 4°C; or snap‐frozen in liquid nitrogen and stored at −80°C until use. Blood samples obtained by cardiac puncture were kept at 4°C for approximately 2 h to promote clot formation. Then, centrifuged at 1200 rpm for 10 min; the serum was collected and stored at −20°C.

### 2.5 Total protein extraction and immunoblots

Frozen visceral adipose tissue was placed in a mortar with liquid nitrogen and macerated. The macerated tissue was collected in Eppendorf tubes containing 500 μL of lysis buffer (20 mM Tris pH 7.4, 137 mM NaCl, 25 mM β‐glycerophosphate pH 7.4, 2 mM PPiNa, 2 mM EDTA pH 7.4, 1% Tritón X‐100 y 10% glycerol), supplemented with the “Complete” proteases inhibitors cocktail (Roche) and phosphatase inhibitors (200 mM Na_3_VO_4_, 0.1 mM DTT, 1 mM PMSF). After 10 min incubation on ice, samples were centrifuged at 14,500 rpm at 4°C, the supernatants were recovered and stored at −80°C. Immunoblot was performed as previously described.^15^ The immune interactions were visualized by chemiluminescence using a LI‐COR Biosciences instrument. Densitometry was performed using the Image Studio Software Version 5.2.5.

### 2.6 ELISA

IL‐1β, TNF, IL‐6, and IL‐10 serum levels were quantified using ELISA MAX^TM^ Deluxe Sets from Biolegend, following the manufactures instructions.

### 2.7 Lipids determination

Fasting serum total cholesterol (CHOL) and triglycerides (TG) were determined by an enzymatic colorimetric assay using glucose oxidase test and cholesterol oxidase, 4‐aminophenazone (Roche‐Cobas C111 kit, Roche Diagnostic USA). High‐density lipoprotein cholesterol (HDL‐C) was measured using enzymatic direct methods: PEG cholesterol esterase and PEG cholesterol‐oxidase (HDL‐ C plus 3er generation, Roche‐Cobas C111 kit, Roche Diagnostic USA). Low‐density (LDL) and very low‐density VLDL cholesterol were calculated by Anandaraja's formula.^16^

### 2.8 Adipose tissue histochemistry

Fixed visceral adipose tissue embedded in paraffin was sectioned (5 μM‐thick) and stained with hematoxylin and eosin. Analysis of adipocyte histology and the quantification of the crown‐like structures was performed under light microscopy.

### 2.9 Bioinformatics analysis for proteases cutting sites in pro‐IL‐18

The predictive analysis for potential caspases and proteases cutting sites on the pro‐IL‐18 protein sequences was performed using both human and mouse pro‐IL‐18 sequences (UniProt Q14116 and P70380, respectively) and the webtool SitePrediction http://www.dmbr.ugent.be/prx/bioit2-public/SitePrediction/. The predicted cutting sites shown have a cutting specifity >99%.

### 2.10 Statistical analysis

The results are presented as the mean ± standard deviation of the mean. The data were subjected to two‐way ANOVA or one‐way ANOVA, as indicated, using graph Prism 9. Significant differences were considered with a *p*  < .05.

## 3 RESULTS

### 3.1 Caspase‐1 is dispensable for the control of energy consumption in Balb/c mice

**Figure 1:**
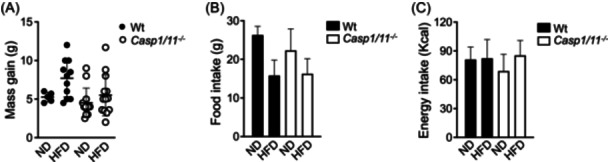
Caspase‐1 is dispensable for energy consumption in Balb/c mice. Eight weeks old wild‐type (Wt) and caspase1/11 deficient (*Casp1/11* ^−/−^) Balb/c mice were fed with regular chow (ND) or with a high‐fat diet (HFD) for 3 months. Mass gain (A), food intake (B), and energy intake (C) were calculated. Data represent the food or Kcal consumed per mouse in a week. Data were analyzed by two‐way ANOVA. The standard deviation of the mean is shown in the graphs.

The Nlrp1b1 inflammasome, which promotes IL‐18 production in adipose tissue and favors lipolysis, protects obese mice from metabolic alterations.^7^, ^12^ We observed that obese C57BL/6 transgenic mice expressing the Nlrp1b1 inflammasome exhibited elevated IL‐18 levels but reduced caspase‐1 activity compared to obese C57BL/6 wild‐type mice, indicating that Nlrp1b1‐mediated IL‐18 production in adipose tissue is caspase‐1 independent. To investigate this further, we generated caspase‐1/caspase‐11 deficient Balb/c mice (Balb/c‐caspase1/11 deficient mice) by backcrossing C57BL/6 caspase‐1/caspase‐11 knockout mice with Balb/c mice for ten generations. We found that eliminating caspase‐1 and caspase‐11 did not affect basal glucose levels, glucose tolerance, and insulin sensitivity in mice fed a regular chow diet (Supplementary Figure 1). Furthermore, when fed an HFD, both wild‐type Balb/c mice and Balb/c‐caspase1/11 deficient mice showed no significant differences in body weight gain (Figure 1A) or caloric intake (Figure 1B,C), indicating that caspase‐1 is dispensable for the control of energy consumption in Balb/c mice.

### 3.2 The Nlrp1b1 inflammasome's protective effect on glucose metabolism is caspase‐1 independent

**Figure 2:**
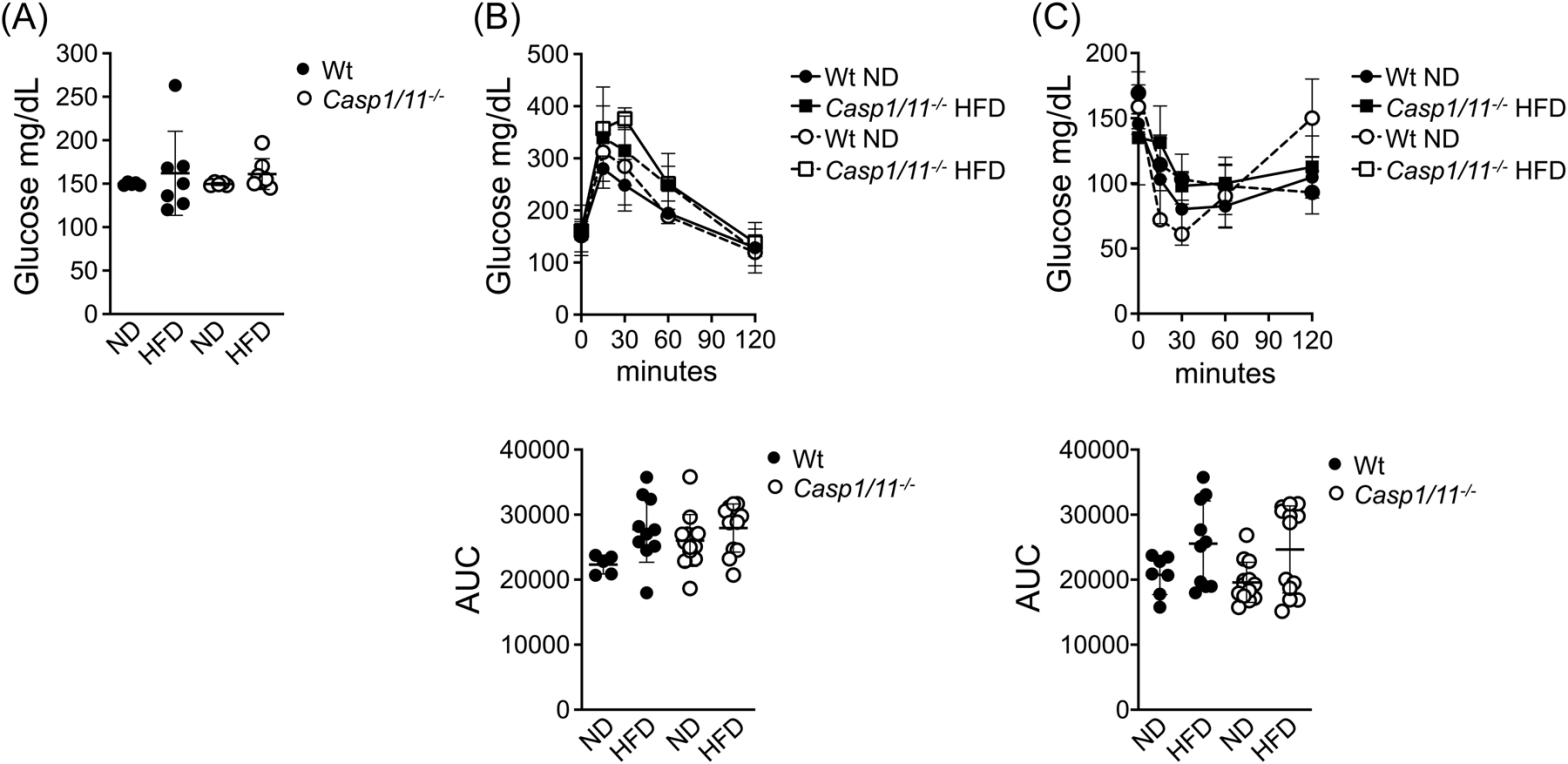
The Nlrp1b1 inflammasome protective effect on glucose metabolism is independent of caspase‐1 activation. Eight weeks old wild‐type (Wt) and caspase1/11 deficient (*Casp1/11* ^−/−^) Balb/c mice were fed with regular chow (ND) or with a high‐fat diet (HFD) for 3 months. (A) Mice were starved for 6 h, and basal blood glucose levels were determined as described under material and methods. (B) For the glucose tolerance tests, starved mice received 1.8 mg of d‐glucose per gram of body mass via intraperitoneal and glucose levels were determined at different time points after glucose administration. The area under the curve (AUC) is shown (lower panel). (C) For the insulin resistance tests, after 6 h of starvation, mice received insulin (1 mU/gr of body mass) via intraperitoneal and glucose levels were determined at different time points after insulin administration. The AUC is shown (lower panel). Data were analyzed by two‐way ANOVA. The standard deviation of the mean is shown in the graphs.

To assess the impact of the HFD on glucose metabolism, we measured basal glucose levels and performed glucose and insulin tolerance tests in fasted mice. Wild‐type Balb/c mice fed an HFD exhibited normal basal glucose levels (Figure 2A), glucose tolerance (Figure 2B), and insulin sensitivity (Figure 2C) compared to mice fed a normal diet, consistent with previous reports. Strikingly, Balb/c‐caspase1/11 deficient mice showed no significant differences in these metabolic parameters when fed an HFD (Figure 2A–C), indicating that the protective effect of the Nlrp1b1 inflammasome on glucose metabolism is independent of caspase‐1 activation.

### 3.3 IL‐18 production in the adipose tissue in response to an HFD is caspase‐1 independent

**Figure 3:**
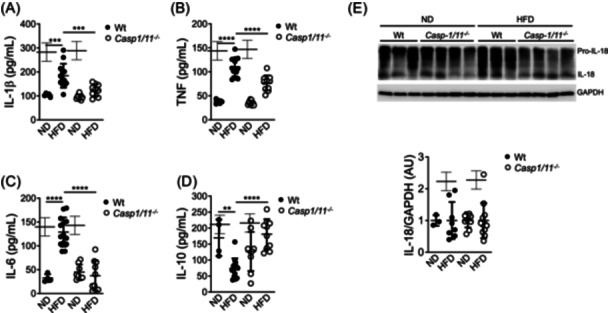
IL‐18 production in the adipose tissue of mice fed with a normal diet or a high‐fat diet is caspase‐1 independent. Eight weeks old wild‐type (Wt) and caspase1/11 deficient (*Casp1/11* ^−/−^) Balb/c mice were fed with regular chow (ND) or with a high‐fat diet (HFD) for three months. Circulating IL‐1β (A), TNF (B), IL‐6 (C), and IL‐10 (D) levels were evaluated by ELISA as described under material and methods. Levels of mature IL‐18 (E) were evaluated by immunoblot using adipose total cell extracts and specific antibodies. GAPDH levels were used as a loading control. Normalized IL‐18/GAPDH densitometry values are shown (E, lower panel). Data were analyzed by two‐way ANOVA. The standard deviation of the mean is shown in the graphs. **p < .01, ***p < .001, ****p < .0001.

Previous studies have shown that the inflammatory process in adipose tissue, initiated by caspase‐1 activation and IL‐1β production in response to energy excess, contributes to glucose intolerance and insulin resistance in obese mice.^4^ Surprisingly, although wild‐type Balb/c mice did not exhibit alterations in glucose metabolism when fed an HFD compared to mice fed a normal diet (Figure 2), they showed increased levels of IL‐1β, TNF, and IL‐6, and reduced levels of IL‐10 in the circulation (Figure 3A–D). This inflammatory profile was found to be dependent on caspase‐1 activation, as HFD‐fed Balb/c‐caspase1/11 deficient mice displayed IL‐1β and IL‐6 levels similar to those observed in mice fed a normal diet (Figure 3A,C). Although TNF levels were reduced compared to HFD‐fed wild‐type Balb/c mice (Figure 3B), they were still higher than those observed in mice fed a normal diet. Similarly, IL‐10 levels in the circulation of HFD‐fed Balb/c‐caspase1/11 deficient mice were not reduced compared to mice fed a normal diet (Figure 3D). Consistent with these findings, HFD‐fed Balb/c‐caspase1/11 deficient mice exhibited reduced immune cell infiltration in the adipose tissue compared to HFD‐fed wild‐type Balb/c mice (Supplementary Figure 2A,B). Overall, these results indicate that wild‐type Balb/c mice develop a mild caspase‐1‐dependent inflammatory response when fed an HFD but are protected from developing glucose metabolic alterations.

In contrast to the caspase‐1‐dependent inflammatory response, the levels of IL‐18 in the adipose tissue of Balb/c‐caspase1/11 deficient mice were similar to those in wild‐type Balb/c mice, regardless of the diet (Figure 3E, upper and lower panels). These findings, along with our previous data,^12^ suggest that IL‐18 production in the adipose tissue, mediated by the Nlrp1b1 inflammasome, is independent of caspase‐1. This highlights the caspase‐1‐independent role of the Nlrp1b1 inflammasome in IL‐18 production and its potential protective role in obesity.

### 3.4 Caspase‐1‐mediated lipid alterations are independent of IL‐1β and IL‐18 production in mice fed with an HFD

**Figure 4:**
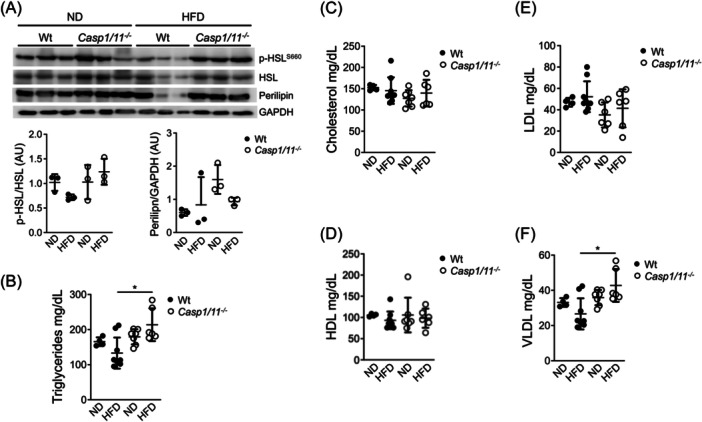
Caspase‐1 deficicy promotes lipid alterations in Balb/c mice fed with a high‐fat diet. Eight weeks old wild‐type (Wt) and caspase 1/11 deficient (*Casp1/11* ^−/−^) Balb/c mice were fed with regular chow (ND) or with a high‐fat diet (HFD) for 3 months. Levels of phosphorylated HSL at serine residue 660 (p‐HSL^s660^), total HSL, and Perilipin levels ([A] upper panel) were evaluated by immunoblot using adipose total cell extracts and specific antibodies. GAPDH levels were used as a loading control. p‐HSL^s660^/HSL ([A] lower left panel) and Perilipin/GPADH (Lower right panel) densitometry values are shown. Circulating Triglycerides (B), Cholesterol (C), and HDL (D) levels were quantified in the plasma from wild‐type (Wt) and caspase1/11 deficient (*Casp1/11* ^−/−^) Balb/c mice as indicated under material and methods. LDL (E) and VLDL (F) levels were calculated using Anandaraja's formula as described under materials and methods. Data were analyzed by two‐way ANOVA. The standard deviation of the mean is shown in the graphs. *p < .05.

Previous studies have shown that IL‐18 promotes lipolysis and has beneficial effects on glucose metabolism in obese mice.^7^, ^12^ In line with this, we examined the levels of active hormone‐sensitive lipase (HSL), a key enzyme involved in lipolysis, and perilipin, a protein associated with lipid droplets, in the adipose tissue of wild‐type and caspase1/11 deficient Balb/c mice. Interestingly, we found no differences in the levels of active HSL or perilipin between the two genotypes, regardless of the dietary regimen (Figure 4A, upper and lower panels). This suggests that the absence of caspase‐1 does not impair HSL activity which correlates with the presence of mature IL‐18 in the adipose tissue.

Next, we assessed lipid levels in the circulation of fasted wild‐type and Balb/c‐caspase1/11 deficient mice. Unlike the alterations observed in caspase‐1 deficient C57BL/6 mice, we found that the levels of triglycerides, cholesterol, HDL, LDL, and VLDL were similar between wild‐type Balb/c mice and Balb/c‐caspase1/11 deficient mice fed a normal diet (Figure 4B–F). Moreover, HFD‐fed wild‐type Balb/c mice did not show significant changes in lipid levels compared to mice fed a normal diet (Figure 4B–F). However, the absence of caspase‐1 in animals fed with the HFD promotes lipid alterations in fasted animals as observed in triglycerides (Figure 4B) and VLDL levels (Figure 4F) compared to HFD‐fed wild‐type Balb/c mice. This is in agreement with previous results reporting that the absence of caspase‐1 in fasted animals promotes TG and VLDL in circulation.^17^ These findings suggest that caspase‐1 is necessary to control triglyceride and VLDL levels in response to lipid excess in Balb/c mice. It is worth noting that caspase‐1 has been implicated in regulating lipid metabolism in C7BL/6 mice, separate from its role in IL‐1β and IL‐18 maturation and production.^18^

## 4 DISCUSSION

Obesity, caused by an imbalance between energy intake and expenditure, contributes to the development of various conditions such as diabetes, vascular and heart problems, dementia, and cancer. The primary driver of obesity‐related diseases is the chronic inflammatory process initiated in the adipose tissue due to excessive lipid accumulation.^1^

Inflammatory cytokines like IL‐1β, TNF, and IL‐6 activate the JNK and IKK inflammatory pathways in metabolic tissues such as the liver, muscle, and brain, which impair insulin signaling. This results in reduced glucose uptake, decreased energy expenditure, and increased food intake.^19^ Consequently, lipid accumulation in the adipose tissue perpetuates the inflammatory process.

The presence of excess lipids triggers the formation of the inflammasome, specifically the Nlrp3 inflammasome, which leads to the recruitment and activation of caspase‐1. Active caspase‐1 then facilitates the processing and maturation of pro‐IL‐1β, initiating the inflammatory response in the adipose tissue.^5^ Although increased levels of IL‐18 have also been observed in the circulation of obese mice and humans, recent evidence suggests that, unlike IL‐1β, IL‐18 plays a protective role in the metabolism of both healthy and obese individuals. IL‐18 promotes energy expenditure and regulates food intake.^8^, ^9^ Furthermore, it has been recently discovered that the Nlrp1b1 inflammasome, rather than the Nlrp3 inflammasome, mediates IL‐18 maturation in the adipose tissue.^7^

Interestingly, our findings demonstrate that caspase‐1 and caspase‐11 are not essential for IL‐18 production in adipose tissue. Balb/c mice deficient in caspase‐1 and caspase‐11 exhibit detectable levels of mature IL‐18, regardless of their diet. However, our data support the notion that caspase‐1 is required for IL‐1β production in the adipose tissue in response to a HFD.^5^, ^20^

Considering that the Nlrp1b1 inflammasome is responsible for IL‐18 processing while the Nlrp3 inflammasome is required for IL‐1β maturation, our findings indicate that in the adipose tissue, two inflammasome complexes are formed: i) the Nlrp3‐Asc‐Caspase‐1 complex responsible for IL‐1β production, and ii) the Nlrp1b1‐Asc complex, along with an unidentified caspase, responsible for IL‐18 production; and that the Nlrp1b1 inflammasome is active under homeostatic and metabolic stress conditions to maintain IL‐18 base levels while the Nlrp3 inflammasome is triggered by abnormal energy excess to release mature IL‐1β.

**Figure 5:**
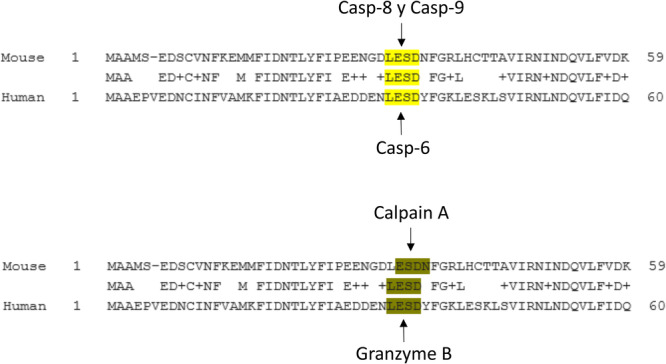
pro‐IL‐18 protein is predicted to be processed by caspases 6, 8, and 9, and by proteases Calpain A and Granzyme B. The human and mouse pro‐IL‐18 sequences were analyzed by the web toolSitePrediction. Different cutting sites were predicted. From these, we selected cutting sites conserved in the human and mouse pro‐IL‐18 protein sequence that showed a cutting specificity >99%. The caspases cleave sites are highlighted in yellow and the proteases cleavage sites are denoted in green.

Through an in‐silico analysis using the web tool “SitePrediction” and human and mouse pro‐IL‐8 protein sequences, we discovered that the conserved caspase‐1 cleavage site in pro‐IL‐18 can also be targeted by Caspase‐6, Caspase‐8, and Caspase‐9 (Figure 5). Interestingly, Caspase‐8 and Caspase‐6 have been proposed to cleave both IL‐18 and IL‐1β. Caspase‐8 activation, triggered by TLR4 and FAS engagement, among other signals, cleaves pro‐IL‐18 into mature IL‐18.^21^, ^22^, ^23^, ^24^ Similarly, Caspase‐6 promotes pro‐IL‐18 maturation in response to Influenza A virus infection.^25^ Together, these findings suggest that the Nlrp1b inflammasome, through caspase‐8 or caspase‐6, mediates IL‐18 maturation in the adipose tissue in response to lipid excess. However, it should be noted that various proteases, rather than caspases, may also participate in IL‐1β maturation.^26^, ^27^, ^28^ Our analysis also revealed Calpain A and Granzyme B as potential proteases involved in IL‐18 maturation, as their predicted cutting sites in pro‐IL‐18 overlap with those in caspase‐1, caspase‐6, and caspase‐8. Further experimental evidence is necessary to determine the specific caspase or protease responsible for IL‐18 production in the adipose tissue both under normal metabolic conditions and metabolic stress.

The role of Asc in IL‐18 production in the adipose tissue also remains to be determined, considering that the Nlrp1b1 inflammasome, in macrophages, can promote IL‐1β production in response to the anthrax lethal toxin independently of Asc.^29^ Additionally, the fact that the absence of Nlrp1b does not completely abrogate IL‐18 production in the adipose tissue of HFD‐feed mice,^7^ suggests an alternative Nlrp1b1‐independent mechanism leading to IL‐18 maturation. This also requires further investigation.

It has been well documented that Balb/c mice, unlike C57BL/6 mice, do not experience severe metabolic alterations when fed a HFD.^13^, ^30^ Our results indicate that although Balb/c mice develop mild systemic chronic inflammation mediated by caspase‐1 in response to lipid excess, IL‐18 prevents the metabolic alterations resulting from HFD feeding. However, this is not the case for C57BL/6 mice, as they exhibit reduced IL‐18 levels in the adipose tissue in response to lipid excess compared to Balb/c mice.^12^ In line with the role of IL‐18 in energy balance regulation,^8^ wild‐type and Balb/c‐caspase1/11 deficient mice fed an HFD showed the same weight gain and caloric intake as mice fed a regular diet. Furthermore, in addition to its positive effects on energy expenditure and food intake, IL‐18 may attenuate the inflammatory response in the adipose tissue by promoting Treg functions,^31^, ^32^ resulting in reduced immune cell infiltration compared to that observed in the adipose tissue of C57BL/6 mice.

Regarding lipid metabolism, our data indicate that no significant alterations occur when feeding wild‐type Balb/c and caspase‐1/caspase‐11‐deficient Balb/c mice an HFD. However, the increase in triglyceride and VLDL levels in caspase‐1/caspase‐11‐deficient Balb/c mice compared to wild‐type mice when fed an HFD aligns with the notion that caspase‐1 also plays a role in lipid metabolism, in addition to its effect on glucose metabolism.^17^, ^18^ Studies have demonstrated that caspase‐1 is required for intestinal triglyceride absorption and triglyceride secretion by the liver, and these effects are independent of caspase‐1‐mediated cytokine pro‐duction.^17^

In summary, our findings provide the first evidence that IL‐18 maturation in the adipose tissue is independent of caspase‐1 and caspase‐11 activation. Moreover, IL‐18 prevents metabolic alterations resulting from excessive lipid intake by regulating energy intake, despite caspase‐1‐dependent increased systemic IL‐1β levels. These results emphasize the importance of developing a combined therapy targeting caspase‐1 activity while enhancing IL‐18 production to reverse glucose and lipid alterations associated with obesity.

## AUTHOR CONTRIBUTIONS

**Luis Román‐Domínguez**: Formal analysis; investigation; writing—original draft. **Jonathan Salazar‐León**: Formal analysis; investigation; writing—original draft. **Karla F. Meza‐Sosa**: Formal analysis; investigation; writing—review and editing. **Leonor Pérez‐Martínez**: Formal analysis; funding acquisition; writing‐review and editing. **Gustavo Pedraza‐Alva**: Conceptualization; formal analysis; funding acquisition; project administration; supervision; writing—original draft; writing—review and editing.

## CONFLICT OF INTEREST STATEMENT

The authors declare that they have no competing interests.

## ETHICS STATEMENT

The authors have nothing to report.

## Supporting information

Supporting information.

Supporting information.

Supporting information.

## Data Availability

The authors have nothing to report.
