# Correction to “Adipose Tissue IL‐18 Production Is Independent of Caspase‐1 and Caspase‐11”

**DOI:** 10.1002/iid3.70095

**Published:** 2024-12-26

L. Román‐Domínguez, J. Salazar‐León, K. F. Meza‐Sosa, L. Pérez‐Martínez, and G. Pedraza‐Alva, “Adipose Tissue IL‐18 Production Is Independent of Caspase‐1 and Caspase‐11,” *Immunity, Inflammation and Disease* 12, no. 4 (2024): e1241, https://doi.org/10.1002/iid3.1241.

In the “2.3 Glucose and insulin tolerance tests” section of the article, Tai's formula is incorrect. Please find the corrected formula below:

The area under the curve was calculated following Tai's formula:

$$Area=\frac{1}{2}\sum_{i=1}^{n}X_{i}-X_{\left(i-1\right)}(Y_{\left(i-1\right)}+Y_{i}),$$

where *X* = time (0, 15, 30, 60, 120 min), and *Y* = glucose (mg/dL) levels at each timepoint [1].

The authors provided the original raw data, which confirmed that despite the erroneously presented formula, the authors calculations were performed correctly, therefore the experimental results and corresponding conclusions mentioned in the paper remain unaffected.

We apologize for this mistake.
