# Supplementary material for: Adipose tissue IL‐18 production is independent of caspase‐1 and caspase‐11
Source: Immun Inflamm Dis. 2024 Apr 17;12(4):e1241. doi: 10.1002/iid3.1241 (PMC11022623; doi:10.1002/iid3.1241)
Supplement: Supplementary file 1 — Supporting information. [file IID3-12-e1241-s002.pdf]

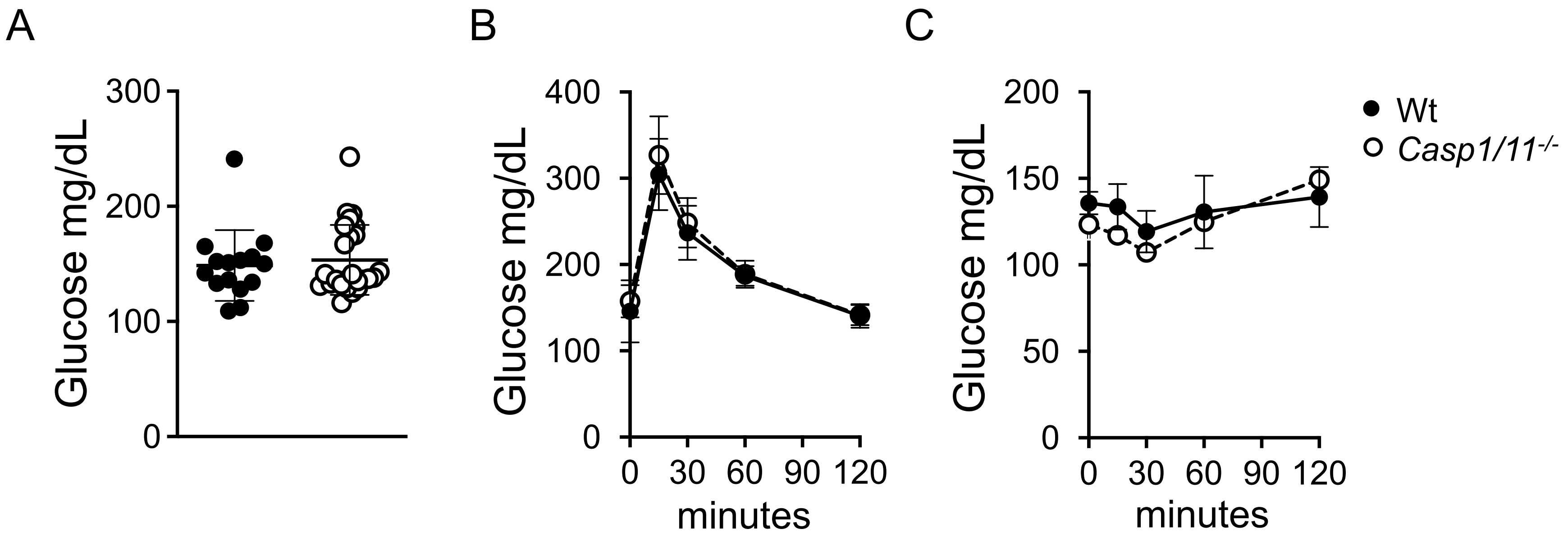

Supplementary figure 1. Metabolic parameters of two moths old wild-type (Wt) Balb/c and caspase1/11 deficient (*Casp1/11*$^{-/-}$) Balb/c mice. At 21 days of age, mice were weaned and fed with regular chaw (normal diet) for 6 weeks. Mice were starved for 6 hours and basal glucose levels were determined (A). Glucose tolerances (B), and insulin resistance (C) tests were performed as described under materials and methods.
