# Supplementary material for: Adipose tissue IL‐18 production is independent of caspase‐1 and caspase‐11
Source: Immun Inflamm Dis. 2024 Apr 17;12(4):e1241. doi: 10.1002/iid3.1241 (PMC11022623; doi:10.1002/iid3.1241)
Supplement: Supplementary file 2 — Supporting information. [file IID3-12-e1241-s001.pdf]

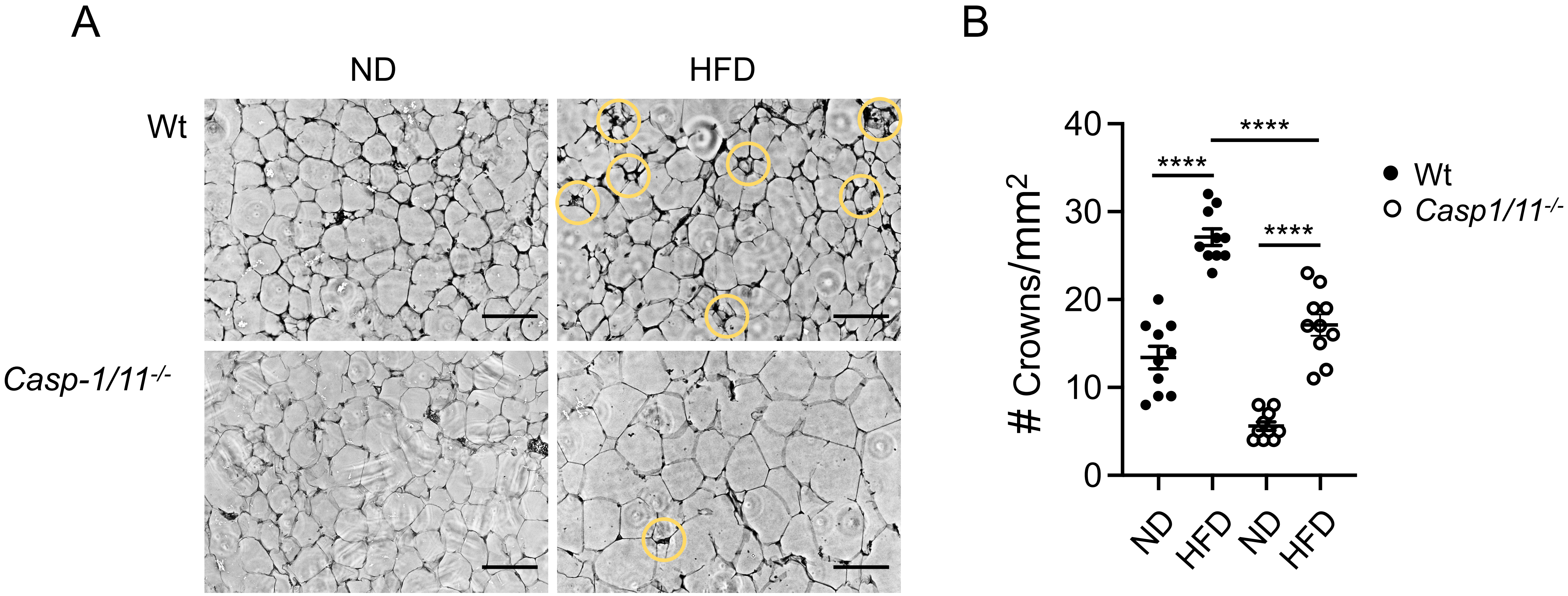

Supplementary figure 2. Wild type (Wt) Balb/c or caspase1/11 deficient (*Casp1/11*$^{-/-}$) Balb/c mice were fed for 3 months with a normal (ND) or with a high-fat diet (HFD). Mice were sacrificed and peritoneal adipose tissue was collected and fixed in paraformaldehyde. A) The cellular infiltration was evaluated in adipose tissue sections stained with hematoxylin and eosin under light microscopy. Yellow circles denote crown-like structures. The bar represents 100 µm. B) Crowns were counted and plotted. Data were analyzed by one-way ANOVA. The standard deviation of the mean is shown in the graphs. **** $p<0.0001$.
