# Supplementary material for: Adipose tissue IL‐18 production is independent of caspase‐1 and caspase‐11
Source: Immun Inflamm Dis. 2024 Apr 17;12(4):e1241. doi: 10.1002/iid3.1241 (PMC11022623; doi:10.1002/iid3.1241)
Supplement: Supplementary file 3 — Supporting information. [file IID3-12-e1241-s003.pdf]

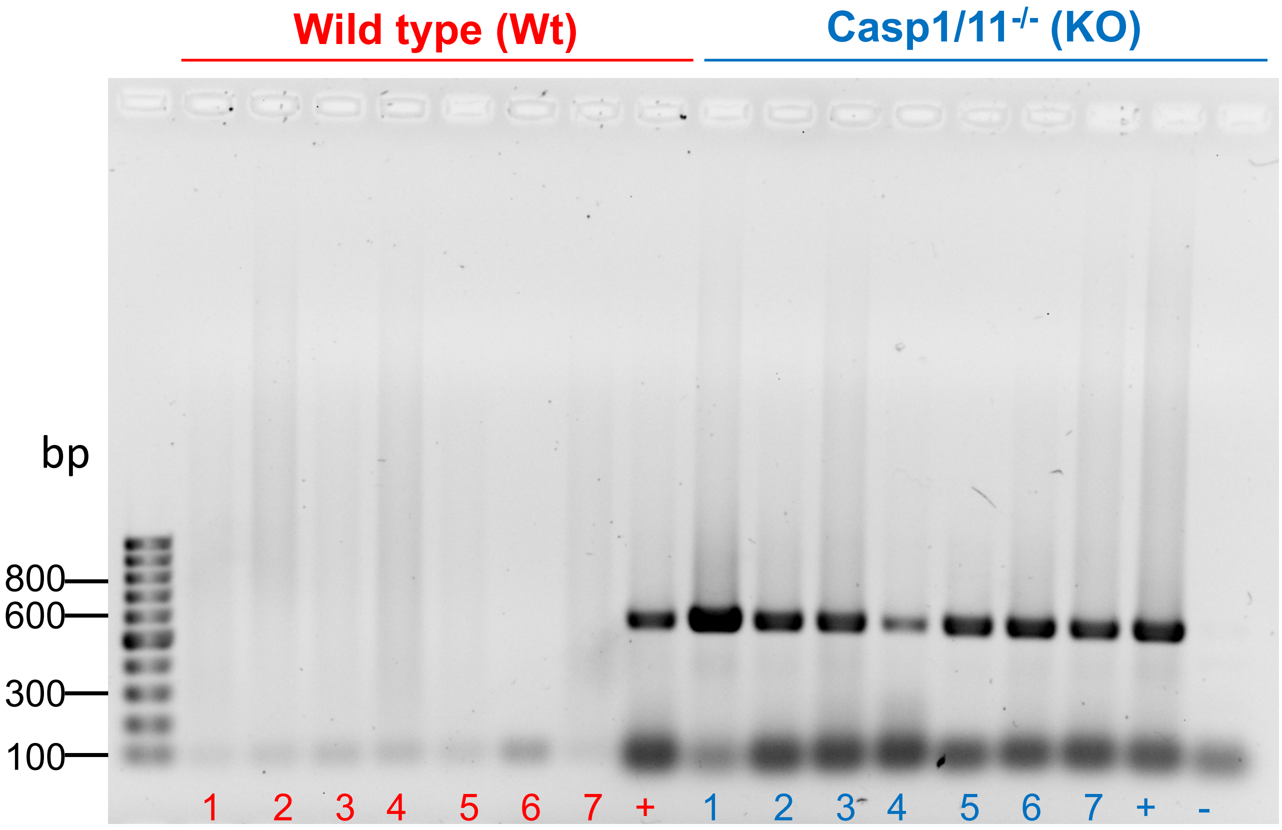

+: positive control (Wt DNA sample + Wt oloigonucleotides)

+: positive control (KO DNA sample) + KO olgonucleotides

-: negative control (Wt DNA sample) + KO oligonucleotides

Wt genotype
Forward primer: MR3769 5’ GAA GAG ATG TTA CAG AAG CC 3’
Revers primer: MR3770 5’ CAT GCC TGA ATA ATG ATC ACC 3’

KO genotype
Forward primer: MR3769 5’ GAA GAG ATG TTA CAG AAG CC
Reverse primer: MR3771 5’ GCG CCT CCC CTA CCC GG 3’

Supplementary Figure 3. *Casp1/11*$^{-/-}$ genotyping. A small biopsy from the tail was collected and genomic DNA was prepared using standard protocols. 50 ng of DNA were used as templated and 10 µM of the indicated Wt or KO oligonucleotides. For PCR reaction DNA was denatured at 95°C for 3 minutes followed by 35 cycle of denaturation (95°C, 30 sec), annealing (66°C, 1 min) and extension (72°C for 2 min) and a final extension of 2 min at 72°C for the Wt allele; and 35 cycle of denaturation (95^{0}C 30 sec), annealing (59°C, 1 min) and extension (72°C for 2 min) and a final extension of 2 min at 72°C for the KO allele. DNA fragments were separated on 1% agarose gel. Wt product size 600 bp; KO product size 630 bp.
